# A matched pilot cohort study of intravenous omadacycline in the treatment of severe pneumonia associated with carbapenem-resistant *Acinetobacter baumannii*

**DOI:** 10.3389/fmicb.2025.1597860

**Published:** 2025-07-23

**Authors:** Dayu Chen, Yechao Chen, Na Hu, Qiaoling Gu, Jingjing Kan, Jinchun Liu, Haixia Zhang

**Affiliations:** ^1^Department of Pharmacy, Nanjing Drum Tower Hospital, Nanjing, China; ^2^Department of Pharmacy, School of Basic Medicine and Clinical Pharmacy, Nanjing Drum Tower Hospital, China Pharmaceutical University, Nanjing, China; ^3^School of Basic Medicine and Clinical Pharmacy, China Pharmaceutical University, Nanjing, China; ^4^Department of Pharmacy, Nanjing Drum Tower Hospital, Nanjing University of Chinese Medicine, Nanjing, China

**Keywords:** omadacycline, tigecycline, carbapenem-resistant *Acinetobacter baumannii*, pneumonia, retrospective matched cohort study

## Abstract

**Background:**

Carbapenem-resistant *Acinetobacter baumannii* (CRAB)-caused severe pneumonia is associated with high mortality rates, and treatment options are limited. Tetracyclines, including tigecycline and omadacycline, have *in vitro* activity against CRAB. We conducted this study to explore the efficacy of omadacycline in CRAB-caused severe pneumonia.

**Methods:**

This retrospective cohort study was performed by collecting data on severe CRAB-caused pneumonia cases treated with omadacycline or tigecycline at Nanjing Drum Tower Hospital from January 2022 to June 2024. A 1:1 propensity score-based matching design based on baseline characteristics was utilized. We compared the incidence of clinical response at day 14 or at the end of treatment (EOT) and other clinical outcomes between the two cohorts.

**Results:**

A total of 40 patients were analyzed, with 20 patients in each cohort. The clinical success rate at day 14 or at the EOT was 65% (13/20) in the omadacycline group compared to 55% (11/20) in the tigecycline group. Both groups had an equal mortality rate, with 8 patients dying within 28 days. Development of tigecycline resistance was observed in one patient. The average duration of invasive mechanical ventilation, vasopressor, renal replacement therapy was also comparable in both groups. Adverse events occurred in 50% (10/20) of patients in the omadacycline group and 75% (15/20) in the tigecycline group, with coagulopathy being significantly lower in the omadacycline group (1/20, 5% vs. 7/20, 35%). Gastrointestinal events were reported in 10% (2/20) of the omadacycline group compared to 30% (6/20) in the tigecycline group. Abnormal liver function was observed in 9/20 patients (45%) in the omadacycline group and 6/20 patients (30%) in the tigecycline group.

**Conclusion:**

This pilot study was the first to explore the efficacy and safety of omadacycline in CRAB-caused pneumonia. Omadacycline demonstrated comparable efficacy to tigecycline in this small pilot study in the treatment of CRAB-caused pneumonia and has a lower incidence of coagulopathy compared to tigecycline, suggesting it may be a viable option, for treating CRAB-caused severe pneumonia, but further prospective research with larger sample sizes is needed to confirm these findings.

## 1 Introduction

*Acinetobacter baumannii*-related severe pneumonia presents a significant mortality rate, estimated to be between 45 and 70%. This pathogen has increasingly garnered interest over the past few decades, primarily due to its extensive antimicrobial resistance (Najafabadi and Soltani, 2024). The implications of *A. baumannii* infections include high morbidity and mortality, as well as increased healthcare costs resulting from prolonged hospital stays and treatment durations. Carbapenem-resistant *Acinetobacter baumannii* (CRAB) is classified as a critical group on the WHO bacterial priority pathogens list. Unfortunately, treatment options for CRAB are limited, especially in resource-limited settings.

Tigecycline is recognized as an effective option for CRAB and is endorsed by guidelines from organizations such as the IDSA (Tamma et al., 2024) and ESCMID (Paul et al., 2022). However, its use is often contingent upon local susceptibility patterns and pharmacokinetic limitations, and it is not always recommended as a monotherapy. Surveillance data from the China Antimicrobial Surveillance Network (CHINET) and the China Antimicrobial Resistance Surveillance System (CARSS) indicate that the resistance rate of *Acinetobacter baumannii* to tigecycline remained below 5% from 2018 to 2022 (Hu et al., 2022). As a result, tigecycline continues to be utilized in China for infections caused by CRAB. Moreover, tigecycline is often incorporated into combination regimens for treating CRAB and carbapenem-resistant *Enterobacteriaceae* (CRE) infections. Following intravenous administration, tigecycline quickly distributes into tissues, resulting in low concentrations in serum and urine; thus, caution is advised when treating infections at these sites. Tigecycline exhibits poor penetration in lung tissue (Rodvold et al., 2006), as indicated by a low tissue-to-serum area under curve ratio of just 2.0, compared to a much higher ratio of 537 for bile (Yang et al., 2021), raising concerns about its effectiveness in treating ventilator-associated pneumonia. As a result, high dosages are often prescribed when using tigecycline for pneumonia (Wang et al., 2023). For CRAB and CRE infections, a high dosage regimen is recommended, typically starting with a 200 mg intravenous loading dose followed by 100 mg administered twice daily.

Omadacycline, a novel antibiotic in the tetracycline class, was approved by the U. S. Food and Drug Administration (FDA) in 2018 (Markham and Keam, 2018) for the treatment of acute bacterial skin and skin-structure infections, as well as community-acquired pneumonia (CAP) (Zhanel et al., 2020). Mechanistically, this antibiotic retains activity against organisms with tetracycline efflux and ribosomal protection genes and does not exhibit cross-resistance with beta-lactams, aminoglycosides, polymyxins, or fluoroquinolones (Chambers, 2019). However, resistance in clinical isolates may still occur through other mechanisms. Compared to doxycycline and minocycline, omadacycline shows significantly greater *in vitro* effectiveness against *Enterobacteriaceae* and *Acinetobacter baumannii*, achieving minimum inhibitory concentrations (MICs) of ≤ 4 μg/mL for 90% of strains, which aligns with the FDA’s susceptibility threshold (Pfaller et al., 2018; Huband et al., 2025). The potential of omadacycline to treat infections caused by multidrug-resistant pathogens, especially CRE and CRAB, remains an important area for further investigation.

However, while omadacycline has demonstrated *in vitro* activity against *Acinetobacter baumannii*, clinical evidence remains limited. A case series reported satisfactory results in five instances where omadacycline was used to treat skin and soft tissue infections caused by extensively drug-resistant (XDR) or multidrug-resistant (MDR) *Acinetobacter baumannii* (Morrisette et al., 2022). Nonetheless, studies involving patients with pneumonia are still lacking. Compared to tigecycline, omadacycline appears to have superior pulmonary penetration, making it a potentially better option for pneumonia treatment (Gotfried et al., 2017). Therefore, this retrospective study collected cases from our center involving the use of omadacycline to treat CRAB pneumonia between January 2022 and June 2024. We conducted an exploratory matched pilot cohort study comparing these cases with those treated with high-dose tigecycline in the same patient population to evaluate the effectiveness of omadacycline in managing CRAB pneumonia.

## 2 Materials and methods

### 2.1 Study design, case definition, and study population

This non-randomized retrospective exploratory pilot cohort study collected patient data from Nanjing Drum Tower Hospital between January 2022 and June 2024. Nanjing Drum Tower Hospital is a large tertiary medical center with three satellite hospitals, totaling approximately 5,000 beds. Patient data were obtained from the hospital information system and microbiology laboratory system. This study complies with the Declaration of Helsinki and has received approval from the Ethics Committee of Nanjing Drum Tower Hospital (2024-723-01). The need for informed consent was waived by the ethics committee due to the observational and retrospective nature of the study.

Adult patients aged 18 years or older were included. Patients were required to meet the diagnostic criteria for severe pneumonia, with CRAB identified as the causative pathogen. The diagnosis of severe pneumonia is based on local and Infectious Diseases Society of America (IDSA) guidelines, which include two major criteria and nine minor criteria. The major criteria include: septic shock requiring vasopressors and respiratory failure requiring mechanical ventilation. The minor criteria are as follows: (1) respiratory rate > 30 breaths/min, (2) PaO_2_/FiO_2_ ratio < 250, (3) multilobar infiltrates, (4) confusion or disorientation, (5) uremia (blood urea nitrogen level > 20 mg/dL), (6) leukopenia (white blood cell count < 4,000 cells/μL), (7) thrombocytopenia (platelet count < 100,000/μL), (8) hypothermia (core temperature < 36°C), and (9) hypotension requiring aggressive fluid resuscitation. To diagnose severe pneumonia, a patient must meet either one major criterion or three or more minor criteria. Patients included in the study were treated with either omadacycline or tigecycline as definitive therapy for CRAB severe pneumonia for at least 72 h. The selection of research drugs was based on observed sensitivity from cultures and the clinical judgment of the attending physician regarding each patient’s condition, with no interventions made. In routine practice, our center adheres to both local and IDSA guidelines, which recommend at least 5 days of antimicrobial therapy. Patients were eligible to discontinue treatment if they met the following conditions: no fever for 48–72 h, no need for supplemental oxygen unless required for pre-existing conditions, and a maximum of one clinical instability factor, including a heart rate >100 bpm, a respiratory rate above 24 bpm, or a systolic blood pressure of 90 mmHg or lower. Patients receiving omadacycline treatment were designated as the treatment group, while those receiving tigecycline were classified as the control group. The standard intravenous dosing regimen for omadacycline is a loading dose of 200 mg over 1 h, followed by a maintenance dose of 100 mg every 24 h over 30 min. The guidelines recommend a high-dose regimen of tigecycline for the treatment of CRAB, consisting of a loading dose of 200 mg followed by a maintenance dose of 100 mg every 12 h (2). Exclusion criteria included: (1) patients who received prior treatment with either omadacycline or tigecycline within 30 days before hospitalization, (2) patients with insufficient clinical data or incomplete medical records, (3) patients with other primary pathogens identified that contributed to pneumonia, thereby confounding the results, and (4) pregnant or lactating women.

In this study, propensity score matching (PSM) was utilized to control for confounding variables and improve group comparability. Covariates influencing treatment assignment were selected based on existing literature and theoretical considerations. The tigecycline group will be matched to the omadacycline group in a 1:1 ratio based on propensity scores of gender, age-adjusted Charlson Comorbidity Index (aCCI) (Kupcha et al., 2022), baseline Pneumonia Severity Index (PSI) (Vaughn et al., 2024), baseline Acute Physiology and Chronic Health Evaluation (APACHE) II score, and baseline Sequential Organ Failure Assessment (SOFA) score (Dellinger et al., 2023). The balance of covariates before and after matching was evaluated using standardized mean differences, targeting values below 0.1 to ensure adequate balance. This approach ensured a rigorous comparison between groups while minimizing the impact of confounding factors.

### 2.2 Outcome measurement and data collection

The primary outcome of this cohort study was clinical success, assessed at day 14 or at the end of treatment (EOT), whichever occurred first. Clinical success was assessed based on the following criteria: (1) survival, (2) resolution of clinical symptoms including any sign of Systemic Inflammatory Response Syndrome (SIRS), detailed definition of SIRS can be found in Table 1 (Bone et al., 1992), (3) significant improvement in radiological findings, (4) enhanced oxygenation and alleviation of respiratory distress, and (5) no requirement for new antimicrobial therapy targeting CRAB within 7 days of the follow-up visit. Failure to meet these criteria constituted clinical treatment failure and was recorded as such. The secondary outcomes included early clinical response, 28-day all-cause mortality, CRAB eradication rate, emergence of treatment-related drug resistance and adverse events during therapy. Early clinical response is defined as a noticeable improvement in clinical symptoms and signs within 4 days following the initiation of treatment. This improvement may include a reduction in fever (body temperature < 37.5°C), improvement in radiological findings, alleviation of respiratory distress, and better physical examination findings, such as clearer lung sounds or diminished wheezing. Furthermore, early clinical efficacy can be evaluated by laboratory markers, including a decrease in inflammatory markers (30% reduction in C-reactive protein levels and white blood cell count, 50% reduction in procalcitonin). The proportion of patients receiving organ support, including invasive mechanical ventilation (IMV), vasopressors, and renal replacement therapy (RRT), as well as the duration of these interventions, was assessed. The adverse events observed during treatment included gastrointestinal events, coagulopathy, abnormal hepatic function, and other adverse events. The detailed definitions of adverse events can be found in the Supplementary Table S1.

**Table 1:** Definition of SIRS (Bone et al., 1992).

Symptoms	Definition
Body temperature	>38°C or <36°C
Heart rate	>90/min
Respiratory function	Respiratory rate >20/min or PaCO_2_ < 32 mmHg
White blood cell count	>12,000/mm^3^ or <4,000/mm^3^ or >10% immature bands

In this cohort study, data collection focused on gathering comprehensive patient information to evaluate the efficacy and safety of omadacycline compared to tigecycline in treating severe pneumonia caused by CRAB. Clinical records of all patients were reviewed and evaluated by two researchers (Yechao Chen and Na Hu). Any disagreements between the reviewers were resolved through consultation with a third reviewer (Dayu Chen) to reach a consensus. The data were extracted from electronic medical records and included several key aspects including demographic data, hospitalization information, infection information, symptoms and clinical indicators of pneumonia, organ support during treatment, laboratory test results, and information of adverse events. Demographic data included age, gender, body mass index (BMI) and aCCI. Hospitalization information included admission and discharge date, intensive care unit (ICU) length of stay, and 30-day readmission.

### 2.3 Statistical methods

Descriptive statistics were used to summarize baseline characteristics of patients, including age, sex, comorbidities, and severity of pneumonia. Categorical variables were compared using the Chi-square test or Fisher’s exact test, as appropriate. Continuous variables were analyzed using *t*-tests or Mann–Whitney *U* tests, depending on data distribution. To compare clinical outcomes between the omadacycline and tigecycline groups, logistic regression analysis was performed, adjusting for potential confounding factors such as age, sex, and comorbidities. A *p* < 0.05 was considered statistically significant. The odds ratio (OR) with a 95% confidence interval (CI) was reported for clinical outcomes. Data analysis was conducted using R software (version 4.4.1). GraphPad Prism 9.2 (GraphPad Software, La Jolla, CA, USA) was used for statistical analysis and plotting graphs. A *post-hoc* power analysis was calculated using PASS 11.0 software and indicated that the study had a power of 0.21 to detect a significant difference in the observed clinical cure rates of the 2 cohorts. This means that the study is underpowered due to the limited sample size. In this study, multivariable logistic regression was used to identify factors associated with treatment failure. Univariate analyses identified variables significantly associated with failure (*p* < 0.05) or deemed clinically relevant, which were then included in the regression model. To address multicollinearity, variance inflation factors were calculated; variables with VIFs exceeding 10 were excluded or combined to reduce collinearity. The model was refined using stepwise forward selection methods.

## 3 Results

### 3.1 Patients

According to the predetermined patient inclusion criteria and exclusion criteria of this study, a total of 20 patients treated with omadacycline and 67 patients treated with tigecycline were included and preliminarily assessed. After 1:1 matching for baseline characteristics, the final analysis retained 20 patients in each group. The patients included in this study had an average age of 59 years and an average aCCI of 4.5, indicating that most were middle-aged or older with underlying comorbidities. The mean (SD) APACHE II score was 21.2 (5.7), and the mean (SD) SOFA score was 9.9 (3.6), while the mean (SD) PSI score was 113.3 (23.6). Among the patients, 10 were classified as PSI Class III, 17 as PSI Class IV, and 13 as PSI Class V. These scores suggest a high severity of pneumonia and infection in the patient cohort. Detailed treatment information of the included patients can be found in Supplementary Table S2. All patients received antimicrobial treatment prior to receiving omadacycline or tigecycline, and each patient was treated with at least two different antimicrobial agents. The primary outcome associated with various combination therapies of antimicrobial agents are summarized in Supplementary Table S3. The detailed baseline characteristics of the patients are described in Table 2. No significant differences in the baseline characteristics was found between treatment group and control group. All patients were administered at least one additional antibiotic alongside either omadacycline or tigecycline.

**Table 2:** Baseline characteristics.

Characteristics	Omadacycline group (*n* = 20)	Tigecycline group (*n* = 20)	*P*-value
Demographics			
Age (years)	58.6 ± 15.1	59.7 ± 14.5	0.816
Sex (male,%)	11 (55)	12 (60)	0.749
Pulmonary diseases (*n*, %)	14 (70)	15 (75)	0.723
Diabetes mellitus (*n*, %)	8 (40)	9 (45)	0.749
Malignant tumors (*n*, %)	3 (15)	2 (10)	>0.999
aCCI	4.8 ± 2.1	4.7 ± 2.0	0.423
Disease severity			
PSI risk class			0.934
III (*n*, %)	5 (25)	5 (25)	
IV (*n*, %)	8 (40)	9 (45)	
V (*n*, %)	7 (35)	6 (30)	
SOFA	10.1 ± 3.6	9.9 ± 3.7	0.667
APACHE II	20.5 ± 5.8	20.9 ± 5.6	0.440
Presence of bacteremia (*n*, %)	4 (20)	3 (15)	
Body temperature (°C)	38.3 ± 1.0	38.2 ± 1.1	0.716
Antibiotics prior to study drugs			
Carbapenems (*n*, %)	15 (75)	14 (70)	0.723
Antipseudomonal β-lactam (*n*, %)	12 (60)	13 (65)	0.744
Fluoroquinolone (*n*, %)	11 (55)	12 (60)	0.749
Macrolides (*n*, %)	6 (30)	6 (30)	>0.999
Vancomycin or linezolid (*n*, %)	18 (30)	16 (30)	0.661
Duration of study drugs			
Mean (days)	11.3	12.9	0.540
Median (days)	12.0	10.0	
Organ support at baseline			
IMV (*n*, %)	14 (70)	15 (75)	0.723
Vassopressors (*n*, %)	13 (65)	10 (50)	0.337
RRT (*n*, %)	11 (55)	9 (45)	0.527

IMV, invasive mechanical ventilation; RRT, renal replacement therapy; SOFA, Sequential Organ Failure Assessment; APACHE, Acute Physiology and Chronic Health Evaluation; PSI, pneumonia severity index; aCCI, age-adjusted Charlson Comorbidity Index.

### 3.2 Efficacy

**Figure 1:**
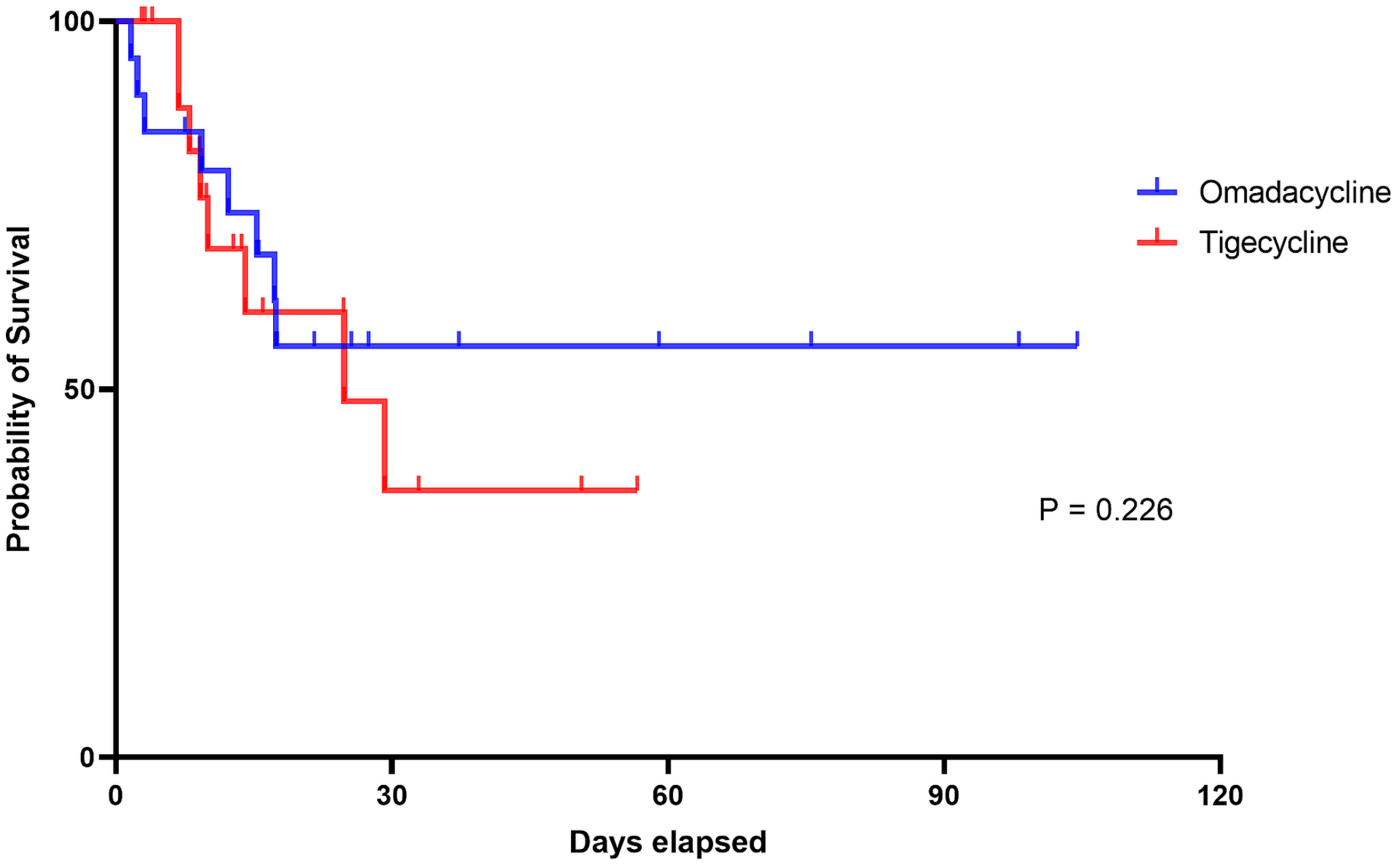
The Kaplan–Meier survival curves of this study. The red line represents the tigecycline group and the blue line represents the omadacycline group. The log-rank test between the two groups indicates no significant difference (*p* = 0.226).

In this study, patients treated with omadacycline exhibited a slightly higher probability of clinical success, with 13 (65%) patients achieving a clinical success by day 14 or at the EOT (Table 3). In contrast, among patients receiving tigecycline, 11 (55%) achieved cure within the same timeframe, although the difference was non-significant (Adjusted OR: 5.24 95% CI: 0.55–49.87, *p* = 0.149). A *post-hoc* power analysis was conducted for the primary clinical outcome, which yielded a power of 0.21. This indicates that the study is underpowered, and therefore, the interpretation of the results should be approached with greater caution. However, the 28-day mortality rate was identical for both treatment groups, with 8/20 (40%) patients in each group dying within 28 days (Adjusted OR: 0.93, 95% CI: 0.25–3.52, *p* = 0.914); all inpatient deaths occurred within this period. Except for one patient treated with omadacycline who died due to a cardiovascular event 12 days after successful treatment cessation, the remaining 15 patients were considered to have died from severe pneumonia. The survival curve is shown in Figure 1, and no difference was found between the two groups. Notably, in the early stages of treatment, most patients experienced some symptom relief, despite the low overall success rate at day 14 or EOT. By the fourth day of treatment, 16 patients in the omadacycline group and 17 in the tigecycline group achieved clinical responses to the treatment, showing decreased fever and improved clinical indicators (Adjusted OR: 3.37, 95% CI: 0.22–10.43, *p* = 0.338). Omadacycline demonstrated a higher eradication rate (6/20, 30% versus 3/20, 15%) for CRAB, although the difference compared to tigecycline was not statistically significant (Adjusted OR: 2.97, 95% CI: 0.78–11.36, *p* = 0.111). Only one patient in the tigecycline group developed treatment-related tigecycline resistance.

**Table 3:** Clinical outcomes.

Clinical outcomes	Omadacycline	Tigecycline	Unadjusted OR (95% CI)	*P*-value	Adjusted OR (95% CI)**	*P*-value
Clinical success at day 14 or at the EOT (*n*, %)	13 (65)	11 (55)	1.52 (0.43–5.43)	0.519	5.24 (0.55–49.87)	0.149
Early clinical response (*n*, %)	16 (80)	15 (75)	1.33 (0.30–5.93)	0.705	3.37 (0.22–10.43)	0.338
28-day all-cause mortality (*n*, %)	8 (40)	8 (40)	1.00 (0.28–3.54)	> 0.999	0.93 (0.25–3.52)	0.914
CRAB eradication rate (*n*, %)	6 (30)	3 (15)	2.43 (0.51–11.51)	0.264	2.97 (0.78–11.36)	0.111
Emergence of treatment-related drug resistance (*n*, %)	0 (0)	1 (5)	–	–*	–	–*
IVM (*n*, %)	17 (85)	18 (90)	0.63 (0.09–4.24)	0.636	0.56 (0.08–4.01)	0.560
IVM duration (mean days)	14.2	13.9	–	0.950*	–	–*
Vassopressor (*n*, %)	16 (80)	14 (70)	1.71 (0.40–7.34)	0.468	1.66 (0.37–7.54)	0.509
Vassopressor duration (mean days)	10.1	13.4	–	0.319*	–	–*
RRT (*n*, %)	12 (60)	11 (55)	1.23 (0.35–4.31)	0.749	1.17 (0.33–4.17)	0.808
RRT duration (mean days)	14.3	15.7	–	0.694*	–	–*
Gastrointestinal event (*n*, %)	2 (10)	6 (30)	0.26 (0.05–1.49)	0.130	0.26 (0.04–1.54)	0.137
Abnormal liver function (*n*, %)	9 (45)	6 (30)	1.91 (0.52–7.01)	0.330	1.85 (0.49–6.99)	0.366
Coagulopathy (*n*, %)	1 (5)	7 (35)	0.10 (0.01–0.89)	0.039	0.09 (0.01–0.86)	0.036
Other (*n*, %)	1 (5)	2 (10)	0.47 (0.04–5.69)	0.556	0.47 (0.04–5.67)	0.551

*The ORs and CIs were not calculated due to the presence of zero events or continuous variables. **The detailed adjusting covariates were demonstrated in Supplementary Table S4.

Regarding organ function support, 14 patients in the omadacycline group initially received IMV, with 3 additional patients requiring it later during treatment. Similarly, 15 patients in the tigecycline group started with IMV, with 3 more beginning this intervention later. The duration of IMV did not differ significantly between the groups after treatment initiation (14.2 vs. 13.9 days). However, the total IMV days within 28 days post-treatment were fewer in the omadacycline group compared to the tigecycline group (193.6 vs. 208.6 days). The proportion of patients using vasopressors or undergoing RRT was similar between the groups. In the omadacycline group, 13 patients initially received vasopressors, compared to 10 patients in the tigecycline group. Ultimately, 16 and 14 patients were treated with vasopressors in the omadacycline and tigecycline groups, respectively. At the start of treatment, 12 patients in the omadacycline group were undergoing RRT, while 9 patients were in the tigecycline group. During treatment, one additional patient in the omadacycline group and two in the tigecycline group started RRT. The average duration of vasopressors (10.1 vs. 13.1 days) and RRT (13.3 vs. 15.5 days) was similar between the two groups. Despite a slightly higher proportion of patients receiving organ support in the omadacycline group, the total durations of vasopressor use (161.1 vs. 183.0 days) and RRT (159.1 vs. 170.4 days) was shorter compared to the tigecycline group, a pattern observed similarly in the duration of invasive mechanical ventilation. Adjusting covariates for clinical outcomes can be found in Supplementary Table S4.

### 3.3 Safety

After treatment began, adverse events occurred in 10 patients (50%) in the omadacycline group and in 15 patients (75%) in the tigecycline group (*p* = 0.191). Among the adverse events, coagulopathy showed the most significant difference in incidence between the two groups. Coagulopathy was reported in 7 patients (35%) treated with tigecycline, while only 1 patient (5%) in the omadacycline group experienced this condition (Adjusted OR: 0.09, 95% CI: 0.01–0.86, *p* = 0.044). The occurrence of gastrointestinal events was also lower in the omadacycline group. In the tigecycline group, 6 patients (30%) experienced gastrointestinal events, whereas only two patients (10%) in the omadacycline group reported such an event (*p* = 0.235). However, the incidence of abnormal hepatic function appeared slightly higher in the omadacycline group compared to the tigecycline group, although this difference was not statistically significant. Nine patients in the omadacycline group developed abnormal liver function, whereas this occurred in 6 patients in the tigecycline group.

### 3.4 Risk factors of treatment failure

A total of 16 patients experienced treatment failure by day 14 or at the EOT, with 7 in the omadacycline group and 9 in the tigecycline group. All patients who failed treatment in the omadacycline group ultimately died. In contrast, among the 9 patients who failed treatment in the tigecycline group, 1 survived, likely due to timely adjustments in the treatment regimen. Although this patient’s pneumonia did not show significant improvement at the specified assessment points in this study, subsequent treatment adjustments were effective. Initially, this patient was treated with a combination of tigecycline and sulbactam. Upon noticing insufficient efficacy, the sulbactam dosage was increased, and polymyxin B was added as adjunctive therapy. Although this patient had an extended hospital stay, he fortunately survived. Nevertheless, analysis of combined antimicrobial therapy regimens showed that those containing sulbactam did not significantly affect treatment outcomes. Logistic multivariable regression analysis revealed that the presence of bacteremia (OR: 16.13, 95% CI: 1.21–213.68, *p* = 0.035) and PSI risk class V (OR: 6.37, 95% CI: 1.04–38.46, *p* = 0.046) are risk factors associated with treatment failure. The analysis results are presented in Table 4.

**Table 4:** Risk factors of treatment failure.

Risk factors	Univariate analysis			Multivariate analysis		
	OR	95% CI	*P*-value	OR	95% CI	*P*-value
Omadacycline group	0.66	0.18–2.35	0.519			
Age > 65 years	2.28	0.56–9.36	0.253			
Male	1.41	0.39–5.13	0.602			
aCCI	1.20	0.90–1.60	0.215			
Baseline pulmonary diseases	2.17	0.48–9.86	0.317			
Diabetes mellitus	1.09	0.30–3.91	0.896			
PSI risk class V	11.67	2.41–56.49	0.002	6.37	1.04–38.46	0.046
SOFA	1.42	1.09–1.84	0.009*	1.35	0.97–1.90	0.079
APACHE II	1.18	1.02–1.36	0.023*			
Bacteremia	13.80	1.46–130.07	0.022	16.13	1.21–213.68	0.035
IMV at baseline	1.24	0.30–5.18	0.773			
Sulbactam-containing therapy	1.09	0.30–3.91	0.896			

*Due to collinearity between SOFA and APACHE II scores, the more predictive SOFA score was selected for inclusion in the final model.

## 4 Discussion

This exploratory pilot cohort study with a small sample is the first to investigate the use of omadacycline for treating severe pneumonia caused by CRAB. The study found that omadacycline showed similar clinical efficacy and a lower rate of coagulopathy compared to tigecycline in this exploratory pilot study. Compared to tigecycline, omadacycline showed a higher patient cure rate, though the difference was not statistically significant, likely due to the small sample size. Both groups had the same number of patients who died within 28 days. However, omadacycline displayed certain safety advantages. The main safety issues with high-dose tigecycline were gastrointestinal reactions (6/20, 30%) and coagulopathy (7/20, 35%), while omadacycline had lower incidences of coagulopathy (1/20, 5%, *p* = 0.039). Notably, omadacycline did not outperform tigecycline in terms of liver injury events, which warrants further investigation in future studies. However, low statistical power (0.21) limits the interpretation and generalizability of all findings. Low power heightens the risk of Type II error, which occurs when the study fails to identify a true difference between the groups. Even if a genuine difference in clinical cure rates exists, the study may lack the statistical power needed to detect it. Therefore, the lack of statistically significant findings does not imply that there is no actual difference. Consequently, the results should be interpreted cautiously due to the study’s exploratory pilot design and its small sample size (Table 4).

Interestingly, this study revealed that many patients exhibited an early clinical improvement; however, the overall efficacy was less than satisfactory. In total, 17 patients in the tigecycline group demonstrated early responses, yet only 11 ultimately achieved clinical cure. Although the cure rate in the omadacycline group appeared slightly higher, it still fell short of ideal levels. This discrepancy may primarily be attributed to the severity of the disease, but it is also related to the characteristics of tetracyclines. Traditionally, bacteriostatic antibiotics are designed to inhibit bacterial growth (Pankey and Sabath, 2004; Nemeth et al., 2015). However, recent research suggests that bacteria can continue to proliferate in the presence of high concentrations of these inhibitors over extended periods, leading to the emergence of unique subpopulations (Gil-Gil et al., 2024), although this may not be generalizable across all infections or patient populations (Barrasa et al., 2024). These variants exhibit multidrug resistance and display heterogeneity, which can be selectively amplified in antibiotic environments. This phenomenon may contribute to the observed pattern where bacteriostatic agents demonstrate significant early efficacy but ultimately achieve suboptimal clinical cure rates, particularly in the treatment of multidrug-resistant infections. The analysis of organ function support in this study presented insights into the performance of omadacycline and tigecycline in critically ill patients. Both groups initiated IMV at similar rates, with 14 patients in the omadacycline group and 15 in the tigecycline group, reflecting that patients included in this study were critically ill. Although the duration of IMV after treatment initiation did not differ significantly, the total days of IMV within the first 28 days post-treatment were somewhat lower in the omadacycline group. This observation raises the possibility that omadacycline may facilitate respiratory recovery in pneumonia, but further research is necessary to confirm this potential benefit (Yang et al., 2025; Davis et al., 2023). Regarding vasopressor and RRT use, the proportions of patients requiring these interventions were comparable between the groups. However, the omadacycline group exhibited somewhat shorter average durations of vasopressor and RRT use, suggesting a potential therapeutic benefit that may facilitate faster recovery of organ function in patients. While these observations provide valuable preliminary insights, the study should be interpreted as exploratory. The potential advantages of omadacycline require further research to clarify these findings and assess their clinical relevance in managing critically ill patients (Fang et al., 2022). Overall, this exploratory pilot study lays the groundwork for future investigations into the efficacy of these agents in similar contexts.

Regarding the efficacy of omadacycline, some studies indicate significant *in vitro* activity against CRAB (Dong et al., 2020), while others report contrary findings (Noel et al., 2021). The varying conclusions from these studies may be attributed to regional differences and variations in CRAB strains isolated from different populations. *In vitro* studies suggest that current dosing regimens show insufficient PK/PD profiles for CRAB efficacy. Nonetheless, a study in China found that omadacycline exhibited good activity against *Acinetobacter baumannii* with MIC values of ≤0.06–8 mg/L (Dong et al., 2020). These conflicting results may be due to the variable resistance mechanisms of *A. baumannii* and regional resistance epidemiology. Therefore, researching omadacycline’s clinical effects on *A. baumannii* is crucial. This exploratory study suggests that omadacycline has some clinical effectiveness in treating CRAB-associated severe pneumonia in China. Research suggests that the efficacy of antibiotics may be influenced by the site of infection. Standard dosing of tigecycline is thought to be insufficient for effective exposure in the lungs, while omadacycline achieves adequate distribution in pulmonary tissues. Tigecycline appears to be ineffective against bacteremia. Although studies have indicated that omadacycline (Sakoulas et al., 2021) and the newer tetracycline antibiotic eravacycline (Felice et al., 2021) show satisfactory efficacy in managing secondary bacteremia, bacteremia remains a risk factor for treatment failure in this study (Wenzler et al., 2021). This study identifies high PSI class and concurrent bacteremia as risk factors for treatment failure, which aligns with findings from preclinical and PK/PD studies of tetracyclines. However, since the study is underpowered and the sample size is small, we should interpret these risk factors with caution. This study also examined the impact of different combination therapy regimens on treatment failure. Although regimens containing sulbactam are recommended by the current guidelines, our analysis found no significant improvement in treatment success rates with these regimens. Additionally, the patient cohort in this study did not include sulbactam-durloactam, a recently approved and effective drug that is not yet available in China. Future research should explore the efficacy of treatment regimens that include sulbactam-durloactam.

This study revealed that patients treated with omadacycline experienced a higher incidence of abnormal hepatic function compared to those in the tigecycline group, although the difference was not statistically significant. This result is consistent with previous clinical trials of omadacycline. In a randomized controlled trial comparing omadacycline to moxifloxacin for the treatment of community-acquired pneumonia, the most frequently reported adverse event in the omadacycline group was also abnormal liver function (Stets et al., 2019). Similarly, in a study on long-term safety and tolerability of omadacycline for the treatment of *Mycobacterium abscessus* infections, abnormal hepatic function was the second most common adverse event leading to treatment discontinuation (Mingora et al., 2023). Additionally, this proportion was higher than reported in previous studies. Two factors may contribute to this observation. First, this study reflects real-world clinical settings and included patients with baseline hepatic impairment, while prior studies typically excluded such individuals. Second, the patients included in this research had a more severe disease condition, which may increase the likelihood of liver function impairment. Importantly, all cases of liver injury in this study were transient and improved after symptomatic treatment. No patients discontinued omadacycline due to liver injury, and the same was true for the tigecycline group. Nonetheless, this trend warrants further investigation, as tetracycline antibiotics may be associated with abnormal hepatic function, even though most events are clinically insignificant. Despite this, omadacycline overall demonstrates good safety. Omadacycline demonstrated satisfactory safety concerning coagulopathy compared to tigecycline. Although coagulopathy associated with tigecycline has limited its clinical use, omadacycline appears to show significant improvement in this regard. A recent meta-analysis confirmed its satisfactory safety profile, revealing no significant differences in the overall incidence of AEs, treatment-related AEs, serious AEs, or drug discontinuation due to AEs. Additionally, the likelihood of diarrhea was lower with omadacycline compared to the control medications. However, the safety data mentioned above are derived from this small sample exploratory study and are not conclusive. Therefore, the interpretation of these results should be approached with caution due to the study’s low statistical power.

The main strength of this study is the use of matched controls, which helps reduce bias. However, the absence of randomization and the small sample size are significant limitations that should be acknowledged. Previously, there were no controlled studies assessing omadacycline’s clinical efficacy in this patient population, with most research focusing on *in vitro* studies of clinically isolated *Acinetobacter baumannii* strains. Morrisette et al. (2022) published a multicenter case series that included a few patients with *A. baumannii* infections; however, these cases were related to skin and soft tissue infections rather than pneumonia.

Nonetheless, this study has several limitations. Firstly, while it included a control group, it was observational and lacked randomization and allocation, which may introduce bias. Secondly, due to the small sample size, the conclusions drawn here should be viewed as preliminary and not definitive. Finally, the study lacks pharmacokinetic analysis, limiting the ability to validate the PK/PD profile of omadacycline in clinical settings. We suggest that future studies should include PK/PD data to validate exposure-response relationships. Future research should build on this study by increasing the sample size and conducting prospective interventional studies. If possible, patient blood samples should be analyzed to investigate the PK/PD profile in clinical contexts.

## 5 Conclusion

In this exploratory pilot cohort study comparing the treatment of severe CRAB pneumonia with omadacycline and tigecycline, omadacycline showed comparable clinical efficacy and lower coagulopathy rates than tigecycline, but conclusions are preliminary due to limited sample size and study power. However, the conclusions of this study are preliminary, and future research should involve prospective randomized controlled and adequately powered studies based on these initial findings.

## Data Availability

The original contributions presented in the study are included in the article/Supplementary material, further inquiries can be directed to the corresponding authors.
