# Supplementary material for: A matched pilot cohort study of intravenous omadacycline in the treatment of severe pneumonia associated with carbapenem-resistant Acinetobacter baumannii
Source: Front Microbiol. 2025 Jul 23;16:1597860. doi: 10.3389/fmicb.2025.1597860 (PMC12325336; doi:10.3389/fmicb.2025.1597860)
Supplement: Supplementary file 1 [file Table_1.docx]

Table S1 Definitions of adverse events

| Adverse events | Definitions |
| --- | --- |
| Abnormal hepatic function | One of the following: serum ALT or AST > 3 times of the UPN or ALP > 2 times of UPN on two separate occasions at least 24 hours apart; total serum bilirubin >50mmol/L along with elevated serum AST, ALT or ALP level; or INR >1.5 with elevated serum AST, ALT or ALP. |
| Acute kidney injury | Serum creatinine increase of more than 26.5 umol/L within 48 hours or 50% increase from the baseline within 7 days or a urine volume less than 0.5 ml/kg/h for 6 hours. |
| Coagulopathy | One of the following: a decrease in fibrinogen levels to below 1.5 g/L, or an increase in APTT value to more than 1.5 times the upper limit of normal, or an INR value exceeding 1.5, or any bleeding events occurred after the treatment initiated. |
| Gastrointestinal events | Vomiting, constipation, nausea, and diarrhea. |
| Thrombocytopenia | Defined as a platelet count of <100 × 10^3^/µL combined with a drop of more than 25% from the baseline count. |
| Other | Any other adverse events observed during and after treatment were documented. Their potential relationship to the therapy was assessed using the Naranjo scale, with scores above 5 indicating a likely relevance. |

ALT, alanine aminotransferase; AST, aspartate aminotransferase; UPN, upper limit of normal; ALP, alkaline phosphatase; INR, international normalized ratio; APTT, activated partial thromboplastin time.
