# Supplementary material for: A matched pilot cohort study of intravenous omadacycline in the treatment of severe pneumonia associated with carbapenem-resistant Acinetobacter baumannii
Source: Front Microbiol. 2025 Jul 23;16:1597860. doi: 10.3389/fmicb.2025.1597860 (PMC12325336; doi:10.3389/fmicb.2025.1597860)
Supplement: Supplementary file 2 [file Table_2.docx]

Table S2 Clinical characteristics of patients treated with omadacycline or tigecycline for CRAB severe pneumonia

| Baseline characteristics | | | | | | | | | Efficacy outcomes | | | | Safety outcomes | | |
| --- | --- | --- | --- | --- | --- | --- | --- | --- | --- | --- | --- | --- | --- | --- | --- |
| Subject ID | Omadacycline/Tigecycline | Age/sex | Malignant tumor | Pulmonary disease | PSI risk class | Duration  (days) | IMV at baseline | Presence of bacteremia | Clinical cure at 14 day or at the end of treatment | Early clinical response at day 4 | 28-day all-cause mortality | Microbiology eradication at the EOT | Gastrointestinal events | Coagulopathy | Abnormal hepatic function |
| 1 | Omadacycline | 59/female | Liver cancer | No | V | 7 | Yes | No | Yes | Yes | No | No | No | No | Yes |
| 2 | Omadacycline | 55/male | No | AECOPD | III | 12 | Yes | No | Yes | Yes | No | No | No | No | Yes |
| 3 | Omadacycline | 59/female | No | Pulmonary bulla | IV | 3 | Yes | Yes | No | No | Yes | No | Yes | Yes | Yes |
| 4 | Omadacycline | 56/male | No | AECOPD | IV | 10 | Yes | No | Yes | Yes | No | No | No | No | Yes |
| 5 | Omadacycline | 68/female | No | ILD | IV | 13 | No | Yes | Yes | Yes | No | No | Yes | No | No |
| 6 | Omadacycline | 53/female | No | AECOPD | IV | 23 | Yes | No | Yes | Yes | No | Yes | No | No | Yes |
| 7 | Omadacycline | 38/female | No | Asthma | V | 10 | Yes | No | Yes | Yes | No | Yes | No | No | Yes |
| 8 | Omadacycline | 45/male | No | No | III | 14 | Yes | No | Yes | Yes | No | No | No | No | No |
| 9 | Omadacycline | 50/female | Gastric cancer | No | III | 7 | No | No | Yes | Yes | No | Yes | No | No | No |
| 10 | Omadacycline | 38/female | No | No | IV | 16 | No | No | Yes | Yes | No | Yes | No | No | No |
| 11 | Omadacycline | 86/male | No | AECOPD | IV | 17 | No | Yes | No | Yes | Yes | No | No | No | No |
| 12 | Omadacycline | 60/female | No | ILD | V | 2 | Yes | No | No | No | Yes | No | No | No | Yes |
| 13 | Omadacycline | 61/male | No | AECOPD | IV | 8 | No | No | Yes | Yes | Yes | No | No | No | No |
| 14 | Omadacycline | 39/male | No | No | V | 14 | Yes | Yes | No | Yes | Yes | No | No | No | No |
| 15 | Omadacycline | 71/female | Prostate cancer | AECOPD | III | 12 | Yes | No | Yes | Yes | No | No | No | No | Yes |
| 16 | Omadacycline | 54/male | No | ILD | V | 9 | Yes | No | No | No | Yes | No | No | No | No |
| 17 | Omadacycline | 83/male | No | AECOPD | III | 13 | Yes | No | Yes | Yes | No | No | No | No | Yes |
| 18 | Omadacycline | 81/male | No | AECOPD | V | 12 | No | No | No | Yes | Yes | Yes | No | No | No |
| 19 | Omadacycline | 39/male | No | No | V | 21 | Yes | No | Yes | Yes | No | Yes | No | No | No |
| 20 | Omadacycline | 77/male | No | AECOPD | IV | 2 | Yes | No | No | No | Yes | No | No | No | No |
| 21 | Tigecycline | 51/male | No | ILD | IV | 6 | Yes | No | No | No | Yes | No | Yes | No | Yes |
| 22 | Tigecycline | 70/male | No | AECOPD | V | 14 | No | No | No | Yes | Yes | No | No | Yes | No |
| 23 | Tigecycline | 70/male | No | AECOPD | III | 9 | Yes | No | Yes | Yes | No | Yes | No | Yes | No |
| 24 | Tigecycline | 65/female | No | AECOPD | III | 14 | No | No | Yes | Yes | No | Yes | No | No | Yes |
| 25 | Tigecycline | 64/male | No | No | V | 41 | Yes | Yes | No | Yes | No | No | Yes | No | No |
| 26 | Tigecycline | 79/female | Colorectal cancer | No | IV | 14 | No | No | Yes | Yes | No | Yes | Yes | No | No |
| 27 | Tigecycline | 73/male | No | AECOPD | V | 7 | No | Yes | No | No | Yes | No | No | Yes | Yes |
| 28 | Tigecycline | 33/male | No | Asthma | III | 14 | Yes | No | No | Yes | Yes | No | Yes | Yes | Yes |
| 29 | Tigecycline | 56/male | No | No | III | 3 | Yes | No | Yes | Yes | No | No | No | No | No |
| 30 | Tigecycline | 61/male | No | AECOPD | IV | 3 | Yes | No | Yes | Yes | No | No | No | No | No |
| 31 | Tigecycline | 63/male | No | ILD | IV | 10 | Yes | No | No | No | Yes | No | No | Yes | Yes |
| 32 | Tigecycline | 55/female | No | Asthma | IV | 4 | Yes | No | Yes | Yes | No | No | No | No | No |
| 33 | Tigecycline | 65/male | Liver cancer | AECOPD | V | 7 | Yes | Yes | No | No | Yes | No | No | No | No |
| 34 | Tigecycline | 31/female | No | No | IV | 13 | Yes | No | Yes | Yes | No | No | Yes | No | No |
| 35 | Tigecycline | 33/male | No | Cycstic fibrosis | IV | 5 | No | No | Yes | Yes | No | No | Yes | No | No |
| 36 | Tigecycline | 54/female | No | No | V | 25 | Yes | No | No | Yes | No | No | No | No | Yes |
| 37 | Tigecycline | 61/male | No | AECOPD | III | 9 | Yes | No | Yes | Yes | No | No | No | No | No |
| 38 | Tigecycline | 75/male | No | AECOPD | V | 9 | Yes | No | No | No | Yes | No | No | Yes | No |
| 39 | Tigecycline | 82/male | No | AECOPD | IV | 29 | Yes | No | Yes | Yes | Yes | No | No | Yes | No |
| 40 | Tigecycline | 53/female | No | ILD | IV | 23 | Yes | No | Yes | Yes | No | No | No | No | No |
