# Supplementary material for: A matched pilot cohort study of intravenous omadacycline in the treatment of severe pneumonia associated with carbapenem-resistant Acinetobacter baumannii
Source: Front Microbiol. 2025 Jul 23;16:1597860. doi: 10.3389/fmicb.2025.1597860 (PMC12325336; doi:10.3389/fmicb.2025.1597860)
Supplement: Supplementary file 3 [file Table_3.docx]

Table S3 Clinical cure rate among various combination therapies of antimicrobial agents treating CRAB-caused severe pneumonia

| Antimicrobial agents | | | Clinical cure at day 14 or at the end of treatment, n (%) |
| --- | --- | --- | --- |
| Tetracycline | Other antimicrobial agents | Number of patients |  |
| Omadacycline | Sulbactam | 6 | 5 (83.3) |
| Omadacycline | Polymyxin B | 10 | 6 (60.0) |
| Omadacycline | Sulbactam+Polymyxin B | 4 | 2 (50.0) |
| Tigecycline | Sulbactam | 10 | 5 (50.0) |
| Tigecycline | Polymyxin B | 6 | 3 (50.0) |
| Tigecycline | Sulbactam+Polymyxin B | 3 | 2 (66.7) |
| Tigecycline | Amikacin | 1 | 1 (100.0) |
