# Supplementary material for: A matched pilot cohort study of intravenous omadacycline in the treatment of severe pneumonia associated with carbapenem-resistant Acinetobacter baumannii
Source: Front Microbiol. 2025 Jul 23;16:1597860. doi: 10.3389/fmicb.2025.1597860 (PMC12325336; doi:10.3389/fmicb.2025.1597860)
Supplement: Supplementary file 4 [file Table_4.docx]

Table S4 Adjusting covariates for different clinical outcomes

| Clinical outcomes | Adjusting covariates |
| --- | --- |
| Clinical success at day 14 or at the EOT | PSI risk class V, Bacteremia |
| Early clinical response | PSI risk class V |
| 28-day all-cause mortality | PSI risk class V, Bacteremia |
| CRAB eradication rate | Bacteremia |
| IVM (n,%) | PSI risk class V |
| Vassopressor (n,%) | SOFA |
| RRT (n,%) | aCCI |
| Gastrointestinal event (n,%) | SOFA |
| Abnormal liver function (n,%) | SOFA |
| **Coagulopathy (n,%)** | SOFA, Bacteremia |
| Other (n,%) | SOFA |
